# The structure of a human translation initiation complex reveals two independent roles for the helicase eIF4A

**DOI:** 10.1038/s41594-023-01196-0

**Published:** 2024-01-29

**Authors:** Jailson Brito Querido, Masaaki Sokabe, Irene Díaz-López, Yuliya Gordiyenko, Christopher S. Fraser, V. Ramakrishnan

**Affiliations:** 1 MRC Laboratory of Molecular Biology, Cambridge, UK; 2 Department of Molecular and Cellular Biology, College of Biological Sciences, University of California, Davis, CA USA; 3 Present Address: Department of Biological Chemistry and Life Sciences Institute, University of Michigan, Ann Arbor, MI USA

**Keywords:** Ribosome, Cryoelectron microscopy

## Abstract

Eukaryotic translation initiation involves recruitment of the 43S pre-initiation complex to the 5′ end of mRNA by the cap-binding complex eIF4F, forming the 48S translation initiation complex (48S), which then scans along the mRNA until the start codon is recognized. We have previously shown that eIF4F binds near the mRNA exit channel of the 43S, leaving open the question of how mRNA secondary structure is removed as it enters the mRNA channel on the other side of the 40S subunit. Here we report the structure of a human 48S that shows that, in addition to the eIF4A that is part of eIF4F, there is a second eIF4A helicase bound at the mRNA entry site, which could unwind RNA secondary structures as they enter the 48S. The structure also reveals conserved interactions between eIF4F and the 43S, probaby explaining how eIF4F can promote mRNA recruitment in all eukaryotes.

## Abstract (web summary)

The authors report the structure of a human 48S translation initiation complex, finding a second molecule of eIF4A at the mRNA entry site, apart from the one present within the cap-binding complex eIF4F. This second entry-site eIF4A may be responsible for unwinding mRNA secondary structure.

## Main

Initiation of translation in eukaryotes involves over 20 different eukaryotic initiation factors (eIFs). The process starts with the assembly of the 43S pre-initiation complex (43S) consisting of the 40S ribosomal subunit bound to initiation factors eIF1, eIF1A, eIF3 and eIF5 and a ternary complex (TC) of eIF2, guanosine 5′-triphosphate (GTP), and methionyl initiator transfer RNA (tRNA_i_^Met^). In parallel, the cap-binding complex eIF4F bound to the 5′ end of messenger RNA recruits the 43S to form the 48S translation initiation complex, which then scans along the mRNA until it encounters a start codon^1–8^.

The cap-binding complex eIF4F consists of a scaffold protein eIF4G, an m^7^G cap-binding protein eIF4E, and a DEAD-box helicase eIF4A^9^. Additionally, eIF4G interacts with poly(A)-binding protein PABP located at the 3′ untranslated region (UTR) of mRNA, which further brings the 3′ and 5′ ends close together. In metazoans, the attachment of the 43S to the mRNA–eIF4F–PABP complex is mediated by a direct interaction between eIF3 and eIF4G^3–5,8,10,11^. Recently, we determined the structure of a human 48S complex during scanning, which revealed the binding site of eIF4F^4^. While the resolution was modest, the structure showed that eIF4F interacts with eIF3e and eIF3k/l located upstream of the 43S, near the mRNA channel exit site. This finding was consistent with a slotting mechanism of mRNA recruitment and explained the presence of a blind spot between the m^7^G cap structure and the recognition of an initiation codon. Nevertheless, the location of eIF4F in the 48S left an unanswered question about how eIF4A that is part of eIF4F behind the scanning 40S subunit could act as a helicase during scanning and actively unwind secondary structure before it enters the mRNA binding channel.

The helicase activity of eIF4A is stimulated by eIF4G and eIF4B^12–14^. In addition, *Saccharomyces cerevisiae* eIF4B probably induces conformational changes in the 40S subunit to facilitate the attachment of the 43S to mRNA. Yet, it is unclear how eIF4B interacts with eIF4A and/or eIF4F on the surface of the 40S subunit. Interestingly, the ATPase activity of yeast eIF4A does not appear to require eIF4G and eIF4E^15^, whereas in mammals recruitment and scanning require the cap-binding complex eIF4F^13^. Thus, it remained uncertain how eIF4A, eIF4B and the eIF4F complex could work together to unwind secondary structure during mRNA recruitment and scanning.

In our prior structure, we used an mRNA that lacked an AUG codon and was shorter than the footprint of the 48S predicted by the structure^4^. In this Article, we capture a later-stage initiation complex of the 48S complex positioned over a start codon, using a longer, more physiological mRNA with a long and structured 5′ UTR, AUG codon and a 3′ UTR ending in a poly(A) tail. Unexpectedly, a second eIF4A helicase is bound at the mRNA entry channel of the 48S complex. This eIF4A helicase interacts with universally conserved eIF3 subunits and is entirely separate from the eIF4F complex positioned at the mRNA exit site on the other side of the 40S subunit. The structure identifies the interaction of eIF4B with the entry site bound eIF4A, providing insight into its role in regulating this second eIF4A rather than eIF4F during scanning. The discovery of a second eIF4A molecule positioned at the point of entry of mRNA into the mRNA binding channel of the 40S subunit resolves many seemingly contradictory data, including the unanswered question of how secondary structure is unwound by the helicase activity of eIF4A during scanning.

## Structure of a human 48S translation initiation complex

**Extended Data Fig. 1:**
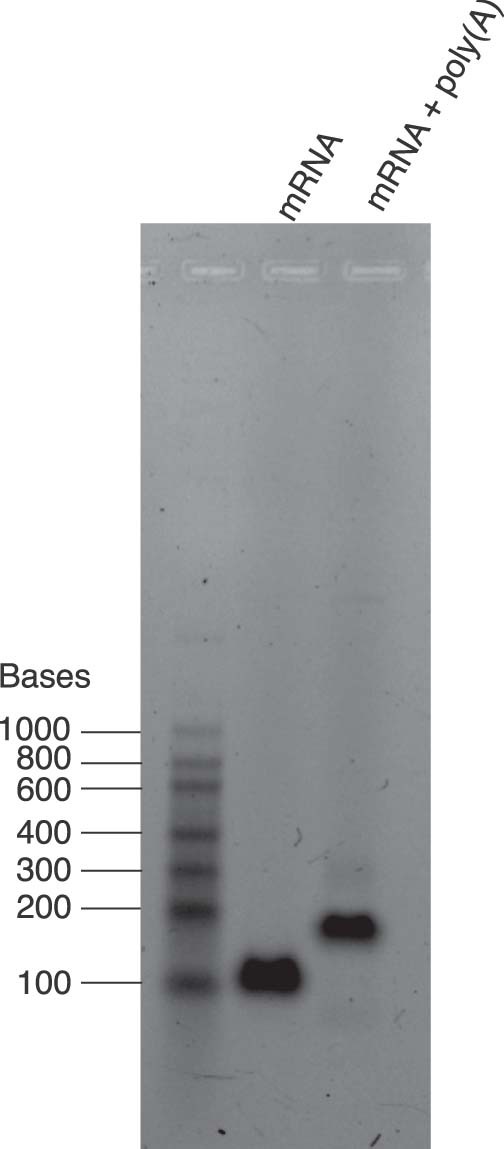
Analysis of capped mRNA after Poly(A) tailing. The polyadenylated mRNA has additional ~90 nucleotides at the 3’ end. The poly(A) tailing was performed according to the protocol provided by the manufacturer (NEB; M0276). The final mRNA, containing a poly(A) stretch, was stored and used to assemble the complex for cryo-EM.

To reconstitute a 48S complex as complete and physiological as possible for cryo-electron microscopy (cryo-EM) analysis, we use a capped mRNA containing a long 5′ UTR (105 nucleotides), an AUG codon, followed by a short coding sequence (9 nucleotides) and a poly(A) tail (~90 nucleotides) (Extended Data Fig. 1). To promote the proposed interaction of the poly(A) tail with the 5′ end of mRNA during complex formation, we included PABP in addition to a full complement of initiation factors. Inclusion of a start codon enables us to generate a late-stage initiation intermediate representing the 48S with the start codon in the P site. To stabilize the complex further, we used Rocaglamide A (RocA), which clamps eIF4A onto polypurine sequences^16–19^, while also including three polypurine motifs (GA)_6_ in the 5′ UTR of the mRNA.

**Fig. 1:**
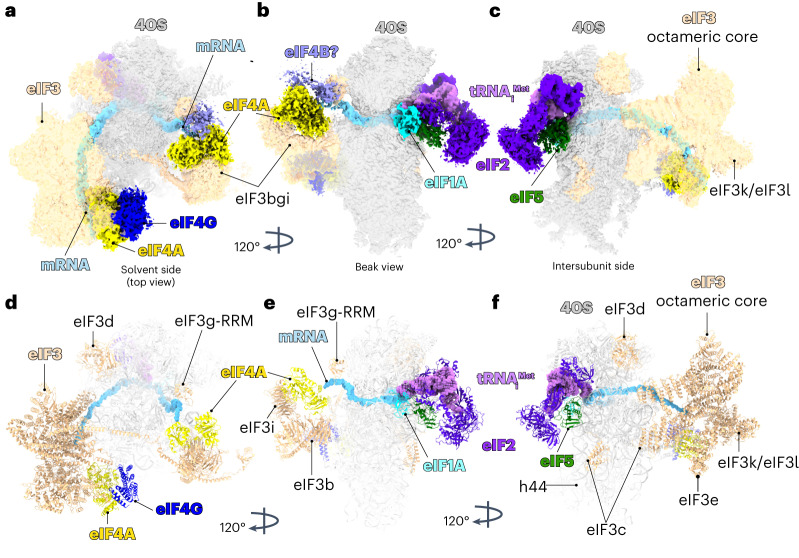
Cryo-EM 3D reconstruction and the structure of a human 48S complex. **a**–**c**, Overview of the cryo-EM density map shown in different orientations. **d**–**f**, Overview of the molecular model of human 48S shown in different orientations. The map contains densities for 40S small ribosomal subunit, eIF3, eIF1A, eIF5-NTD, eIF2, tRNA_i_^Met^, mRNA, eIF4F (eIF4G and eIF4A) and a second molecule of eIF4A bound to eIF3 at the mRNA channel entry site. The map also contains a possible density for eIF4B in contact with the second molecule of eIF4A at the entry site.

**Extended Data Fig. 2:**
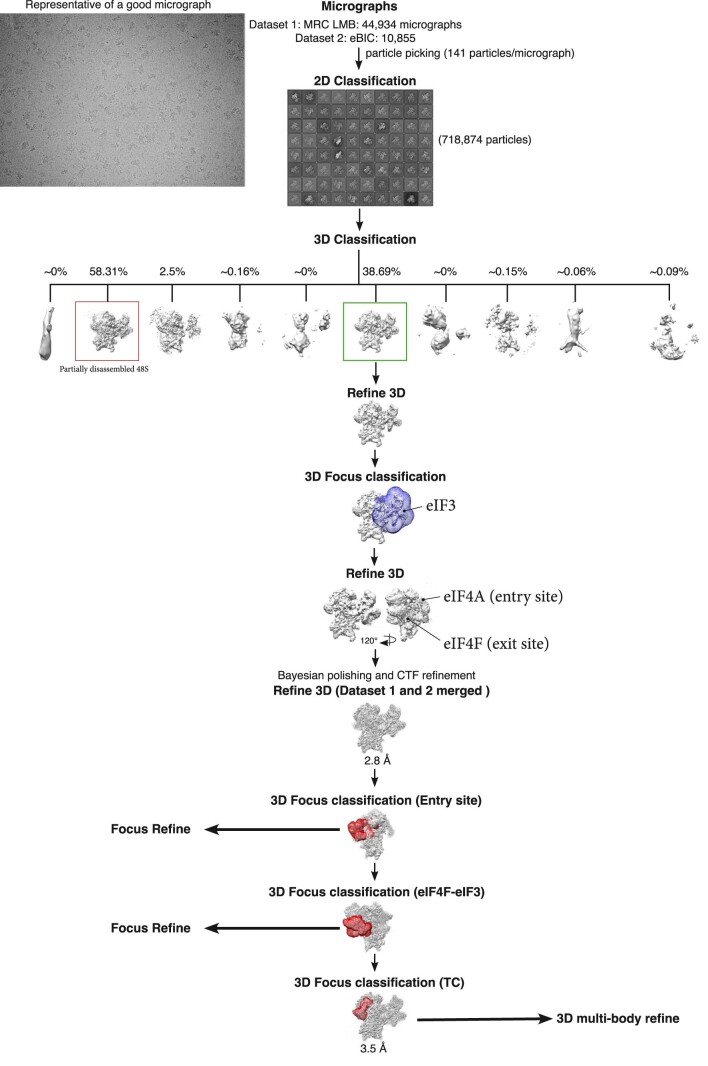
Cryo-EM data analysis of human 48S. We performed two independent data collections, and each dataset (datasets 1 and 2) was analyzed independently. After polishing and CTF refinement, the two datasets were merged, which yielded the 2.9 Å 3D reconstruction of a human 48S.

**Extended Data Fig. 3:**
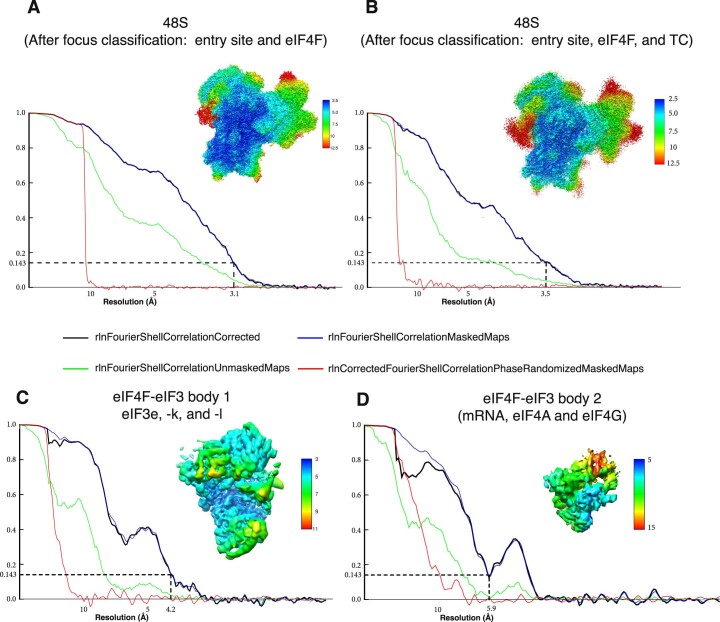
Overall and local resolution. (**a** and **b**) Fourier shell correlation (FSC) curve and local resolution of the 48S complex after focus 3D focus classification at the entry site, eIF4F, and ternary complex. (**c** and **d**) Overall and local resolution of eIF4F-eIF3 after multi-body refinement.

Single-particle reconstruction using cryo-EM reveals density corresponding to the cap-binding complex eIF4F in the same location as seen in our previous structure (Fig. 1 and Extended Data Fig. 2)^4^. Masked classification on the mRNA channel entry site followed by additional masked classification on the region we previously saw for eIF4F yielded a cryo-EM map at 3.1 Å resolution (Extended Data Fig. 3a). Further masked classification on TC yielded a map with a reduced overall resolution of 3.5 Å (Extended Data Fig. 3b) but improved the density for TC.

**Extended Data Fig. 4:**
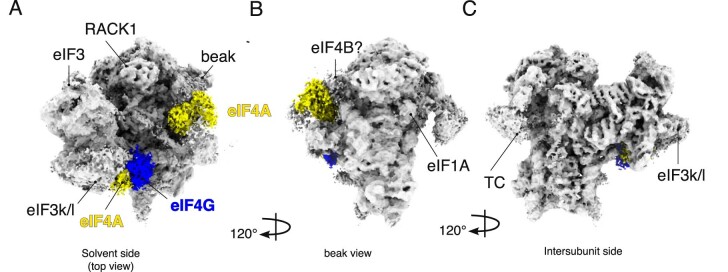
Cryo-EM 3D reconstruction of a human 48S complex assembled without Rocaglamide (RocA). (**a** to **c**) Overview of the cryo-EM density map. The complex contains both eIF4F and the second molecule of eIF4A bound at the mRNA channel entry site. To assembly this complex, we used an mRNA with highly stable RNA stem-loop structure (DLP) located 83 nucleotides downstream of the cap. This mRNA also has an AUG start codon.

We observe additional density extending out from the eIF3bgi module at the mRNA channel entry site (Fig. 1). This density was not present in any previous structures of 48S complexes. The local resolution and detailed shape indicate that this additional density is due to a second eIF4A helicase bound to mRNA, in addition to the eIF4A that is part of eIF4F on the other side of the 40S subunit (Fig. 1). To test whether the presence of this second, entry-site eIF4A is a result of its being trapped by RocA, we determined the structure of a complex without RocA and found that both eIF4F and the second eIF4A are still present (Extended Data Fig. 4). To assemble this complex, we used a capped mRNA with the 5′ UTR of β-globin (55 nucleotides) followed by a coding sequence (124 nucleotides) containing a GC-rich region located 27 nucleotides downstream of the AUG. This GC-rich region forms a downstream loop (DLP) in the mRNA and has been used to trap eIF4A in the 48S^20^.

The local resolution of 5.9 Å for the cap-binding complex eIF4F after focus refinement (Extended Data Fig. 3c,d) is greatly improved compared to our previous structure^4^, and allows us to see direct interactions not only to additional subunits of eIF3 but unexpectedly to the 40S small ribosomal subunit. The improved density shows secondary structure elements and enables us to accurately model the middle domain of eIF4G (HEAT-1 domain) and eIF4A (Fig. 1). Although the mRNA has an m^7^G cap and a poly(A) tail, we did not see any additional density that could be assigned to eIF4E or PABP.

The overall conformation of the structure is a post-scanning intermediate—the initiator tRNA is inserted completely into the P site (a P_in_ state) where it bases pairs with the start codon, and the 40S subunit mRNA binding channel is in the closed conformation. Additionally, the overall resolution allowed us to identify density corresponding to the 40S, eIF1A, eIF2a, eIF2b, eIF2g, tRNA_i_^Met^, the octameric structural core of eIF3 and its peripheral subunits (b, d, g and i; Fig. 1 and Table 1).

**Table 1:** Cryo-EM data collection, refinement and validation statistics

	#48S IC, (EMDB-17297) (PDB 8OZ0)
**Data collection and processing**	
Magnification	105,000
Voltage (kV)	300
Electron exposure (e^−^ Å^−^^2^)	1.197 (LMB) and 1.2391 (eBIC)
Defocus range (μm)	−1.2 to −3.0
Pixel size (Å)	0.826 (LMB) and 0.829 (eBIC)
Symmetry imposed	C1
Initial particle images (no.)	1,914,949 (LMB) and 422,381 (eBIC)
Final particle images (no.)	241,389
Map resolution (Å)	3.5
FSC threshold	0.143
Map resolution range (Å)	2.5 to 12.5
**Refinement**	
Initial model used (PDB code)	6ZMW
Model resolution (Å)	3.7
FSC threshold	0.5
Model resolution range (Å)	2.6 to 3.7
Map sharpening *B* factor (Å^2^)	−10
Model composition	
Non-hydrogen atoms	121,610
Protein residues	11,916
Ligands	91
*B* factors (Å^2^)	
Protein	48.27
Ligands	5,181
R.m.s.d.	
Bond lengths (Å)	0.011
Bond angles (°)	1.014
Validation	
MolProbity score	1.55
Clashscore	3.45
Poor rotamers (%)	0.13
Ramachandran plot	
Favored (%)	93.80
Allowed (%)	5.99
Disallowed (%)	0.21

FSC, Fourier Shell Correlation.

## A second eIF4A helicase in the 48S

**Fig. 2:**
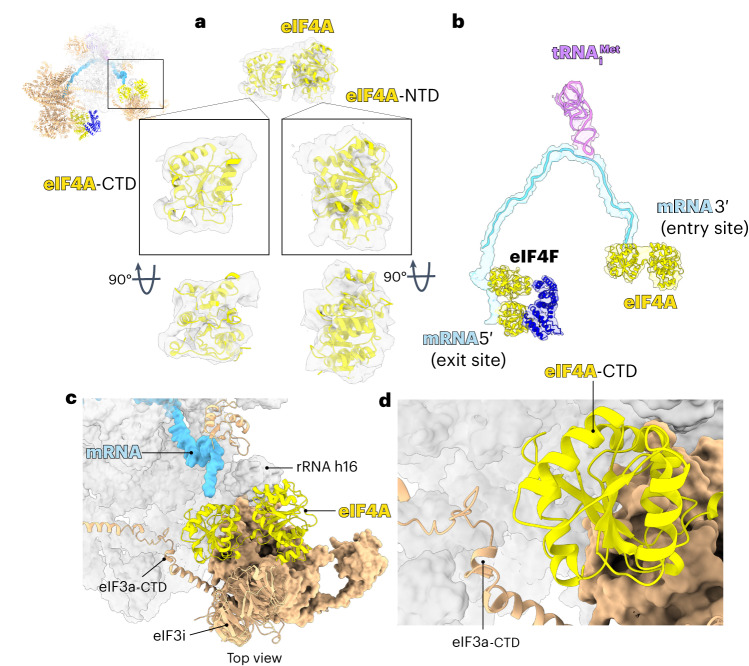
Structure of human 48S reveals a second molecule of eIF4A bound at the mRNA channel entry site. **a**, Atomic model of human eIF4A fitted (correlation 0.9024) into the cryo-EM map filtered to local resolution (6 to 10 Å). **b**, Atomic model of eIF4A, eIF4F, mRNA and tRNA_i_^Met^ fitted into a low-pass filter cryo-EM map. **c**, Interactions of eIF4A at the entry site. **d**, The interface between eIF4A-CTD and eIF3a-CTD.

Compared to our previous 48S structure, we observed additional density at the entry site of the mRNA channel (Fig. 2a,b). Much of this density can be accounted for by eIF4A, which after rigid-body fitting of the known crystal structure of human eIF4A^16^ shows close agreement with the density, especially the eIF4A-CTD, for which almost the entire secondary structure was resolved (Fig. 2b). The density for eIF4A-NTD (where ‘NTD’ is the N-terminal domain) was weaker, presumably because the domain was more disordered. The assignment of this density to eIF4-NTD was dictated by the placement of eIF4A-CTD, the architecture of eIF4A and the orientation of the mRNA (from 5′ to 3′) relative to the ribosome.

eIF4A binds to a pocket formed by eIF3bgi, eIF3a-CTD and ribosomal RNA (rRNA) h16 (Fig. 2c,d). This position of eIF4A agrees well with previous biochemical, cross-link mass spectrometry and proximity labeling data^4,15,21^. Moreover, recent single-molecule data revealed FRET (fluorescence resonance energy transfer) between eIF4A and ribosomal uS19 (ref. ^22^); the latter is located within FRET distance (~97 Å) of this second eIF4A in our structure.

**Extended Data Fig. 5:**
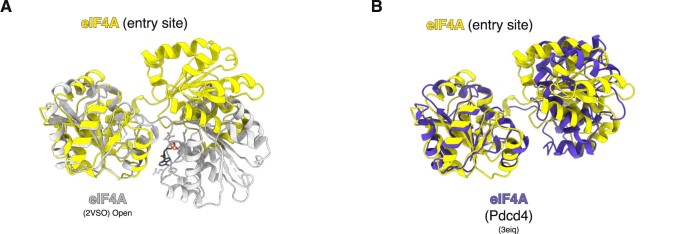
Conformation of molecule of eIF4A bound at the mRNA entry site. (**a**) Superposition of eIF4A with the structure of a yeast eIF4A in complex with eIF4G (PDB:2VSO)^23^. (**b**) Superposition of eIF4A with the structures of eIF4A in complex with Pdcd4 (PDB:3eiq)^24^.

The two RecA domains of the entry site eIF4A adopt an open conformation that differs from that described for the yeast eIF4A–eIF4G complex^23^ (Extended Data Fig. 5). Instead, the conformation of this entry site eIF4A resembles that of the crystallographic structure of the eIF4A–Pdcd4 (Programmed cell death 4) complex^24^ (Extended Data Fig. 5). This conformation allows the NTD and CTD to contact the mRNA while the nucleotide-binding domain adopts a more open conformation. The structure shows that eIF4A could remain bound to mRNA as it cycles through the open and closed conformations during scanning, which may help explain the factor-dependent processivity of human eIF4A described previously^14^.

Unlike previous 48S structures in which mRNA outside the 40S subunit was not seen, there is clear and continuous density for the mRNA, which allowed us to trace its path for the first time from the cap-binding complex eIF4F upstream of the 43S, through the 40S subunit mRNA channel all the way to the second molecule of eIF4A downstream of the 43S (~40 nt upstream and ~20 nt downstream of the AUG; Fig. 2a,b).

It has been shown that yeast 43S stimulates the ATPase activity of eIF4A^15^. Interestingly, this activity of yeast eIF4A does not require eIF4G or eIF4E^15^. Instead, eIF3, especially eIF3i and eIF3g, are required to stimulate the ATPase activity of eIF4A. Because the cap-binding complex eIF4F binds upstream of the 43S, it was unclear how eIF3 subunits apparently downstream of the 43S could affect the activity of eIF4A. However, it has been shown that the eIF3bgi complex is dynamic, binding to both the mRNA entry channel and the subunit interface^25^. Thus, it was not clear whether the eIF3bgi domain stimulates the eIF4A helicase directly or indirectly on the 40S subunit surface. This is now solved by revealing the direct interaction between the second molecule of eIF4A and the eIF3bgi module (Fig. 2). Indeed, the structure reveals that eIF3i is the main binding partner of this second eIF4A.

eIF3a binds to the solvent-exposed site of the 40S, near the mRNA channel exit site. However, its CTD extends toward the solvent side of the 40S, where it interacts with eIF3bgi at the mRNA channel entry site^26^. Our previous cross-linking mass spectrometry indicated that eIF3a-CTD (Lys632) is in close proximity to eIF4A-CTD (Lys291)^4^. Consistent with this observation, the structure shows a close proximity between eIF3a_630–646_, a disordered region in the eIF3a-CTD and Lys291 of eIF4A-CTD (Fig. 2c,d). Indeed, the structure also explains prior biochemical data indicating that eIF3a-CTD and eIF3bgi play important role during recruitment and scanning^27^. This extensive interaction network between eIF4A-CTD and eIF3 can also explain the local resolution disparity between the better-resolved eIF4A-CTD and the NTD. While these interactions stabilize the binding eIF4A-CTD at the entry site, it still allows the highly dynamic transitions between open and closed conformation, which would require a movement of eIF4A-NTD with respect to eIF4A-CTD. This is consistent with a prior single-molecule study suggesting a dynamic transition between open and closed conformation involves a movement of eIF4A-NTD relative to eIF4A-CTD^28^.

**Extended Data Fig. 6:**
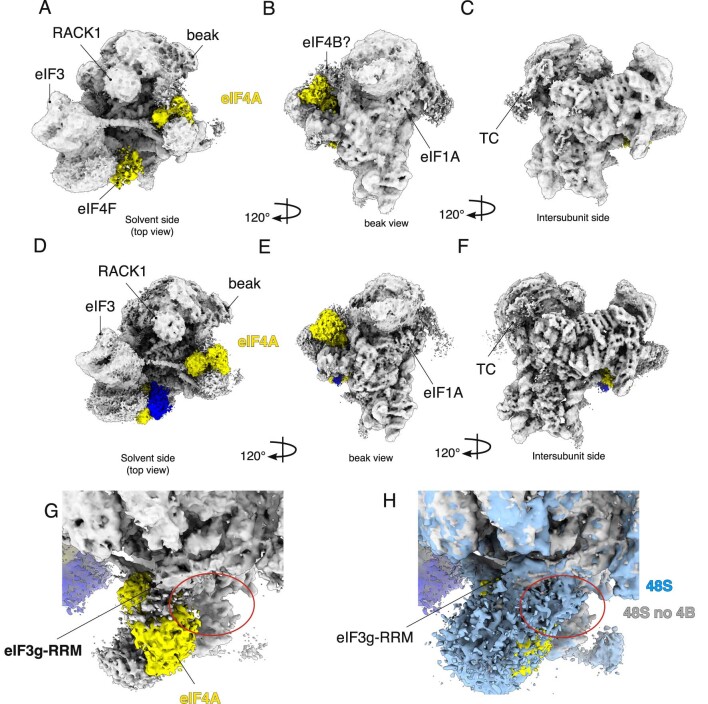
Cryo-EM 3D reconstruction of a human 48S assembled with a truncated eIF4G (residues 557–1137) or without eIF4B. (**a** to **c**) Overview of the cryo-EM density map of a 48S complex assembled with a truncated eIF4G (residues 557–1137). The eIF4A density is visible even without 3D focus classification. The eIF4F density is visible, but is faint without focus classification. (**d** to **f**) Overview of the cryo-EM density map of a 48S complex assembled without eIF4B. The red oval highlights the absence of the density in the complex without eIF4B (**g**) that can be seen in the structure of 48S assembled eIF4B (**h**).

Mammalian eIF4G has two eIF4A-binding domains, one located in the middle domain (HEAT-1) and a second one located at the C-terminal (HEAT-2)^29,30^. The eIF4G HEAT-1 is highly conserved among eukaryotes and is the core of the eIF4F complex, while the eIF4G HEAT-2, not present in *S. cerevisiae*, is poorly conserved and is not essential^29,30^. It has been proposed that HEAT-2 plays a stimulatory role^29^. To test whether HEAT-2 is required for binding of the second molecule of eIF4A, we analyzed a 48S complex with a truncated eIF4G lacking HEAT-2. The second molecule of eIF4A is still present in this complex (Extended Data Fig. 6a–c), suggesting that its binding at the mRNA entry site does not require eIF4G, consistent with previous biochemical data showing that *S. cerevisiae* 43S stimulates the ATPase activity of eIF4A in the absence of eIF4G^15^.

**Fig. 3:**
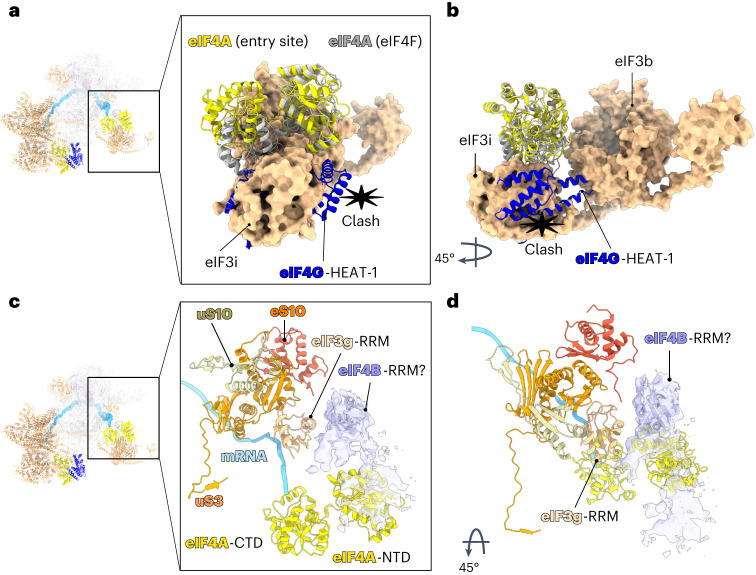
Conformational differences between the two molecules of eIF4A and a possible location of eIF4B in the 48S. **a**,**b**, Superposition of eIF4F with entry site eIF4A to highlight conformational differences shown in in different orientations. eIF4G-HEAT-1 would clash with eIF3i and eIF3b. **c**,**d**, Atomic model of human eIF4B-RRM (PDB: 2J76)^31^ fit into the unassigned cryo-EM density map at the entry site shown in in different orientations. The possible location of eIF4B places it in close proximity to eIF4A-NTD, eIF3g-RRM, uS3, eS10 and uS10.

A previous biochemical study proposed that eIF4F binds at the mRNA entry site^8^. However, at the entry site, the domain of eIF4A that interacts with eIF4G in the eIF4F complex is involved in interactions with eIF3i (Fig. 3a,b). The structure suggests that the position of eIF4A at the mRNA channel entry site is incompatible with its interaction with the eIF4G HEAT-1 seen in the cap-binding complex eIF4F (Fig. 3a,b). We, therefore, propose that this second molecule of eIF4A acts independently of eIF4F.

## A possible location for eIF4B

By itself, eIF4A has low helicase activity. Even though its activity is modulated by the 43S^15^, the helicase activity of eIF4A in scanning the 5′ UTR of mRNA with stable secondary structure is enhanced by the cofactor eIF4B or eIF4H^12,13^. The precise binding site of eIF4B on the 48S remained elusive. Our cryo-EM map contains an unassigned density adjacent to the eIF4A-NTD, in close contact with the eIF3g-RNA recognition motif (RRM) and ribosomal protein uS10 (Fig. 3c,d). Although the resolution of the additional density is low, its size and shape are consistent with the eIF4B-RRM^31^ (Fig. 3c,d). Consistent with this interpretation, the cryo-EM map of a complex formed without eIF4B does not contain this additional density (Extended Data Fig. 6d–h). This location of eIF4B agrees well with previous biochemical data indicating that eIF4B or eIF4H interact with the same region of the eIF4A-NTD^28,32^. Furthermore, previous yeast two-hybrid analysis identified the ribosomal protein uS10, located in close proximity to the unassigned density, to be the main interaction partner of eIF4B^12^. Our data are also consistent with previous cross-linking mass spectrometry data, which revealed close proximity between eIF4B and ribosomal protein uS3^33^. This possible location, which suggests a direct interaction between eIF4B and the entry-site eIF4A, agrees well with the role of eIF4B in increasing the directionality of eIF4A translocation^14^.

In addition to its role as a subunit of eIF4F, we propose that a second molecule of eIF4A at the mRNA channel entry site probably promotes mRNA recruitment and scanning. A second eIF4A in the 48S is also consistent with the finding that eIF4A is in molar excess over other components of eIF4F^34,35^. Nevertheless, even a second eIF4A in the 48S does not account for the substantial excess eIF4A compared to eIF4F in the cell^34,35^. Thus, it is possible that eIF4A could have additional functions apart from its role in the 48S complex. Consistent with this idea, the eIF4A–mRNA interaction has a long lifetime^36^, and eIF4A is known to be able to melt RNA secondary structure in the absence of the 43S^34^.

## eIF4F interacts with eIF3 and ribosomal protein eS7

The recruitment of the 43S to the 5′ UTR of mRNA is a critical step in the translation of eukaryotic mRNAs. Our previous structure of a human 48S complex allowed us to identify the location of eIF4F^4^, but the low local resolution and the adjacent highly flexible region of the 43S meant that we could only infer interactions with non-core subunits of eIF3 (eIF3e, eIF3k and eIF3l). Since these subunits do not exist in *S. cerevisiae*, it was also not clear how eIF4F could interact with the yeast 43S complex. The improved local resolution in this region allows us to build eIF4F more accurately and identify its interactions with the 43S in far greater detail, including additional ones that are probably universally conserved.

**Fig. 4:**
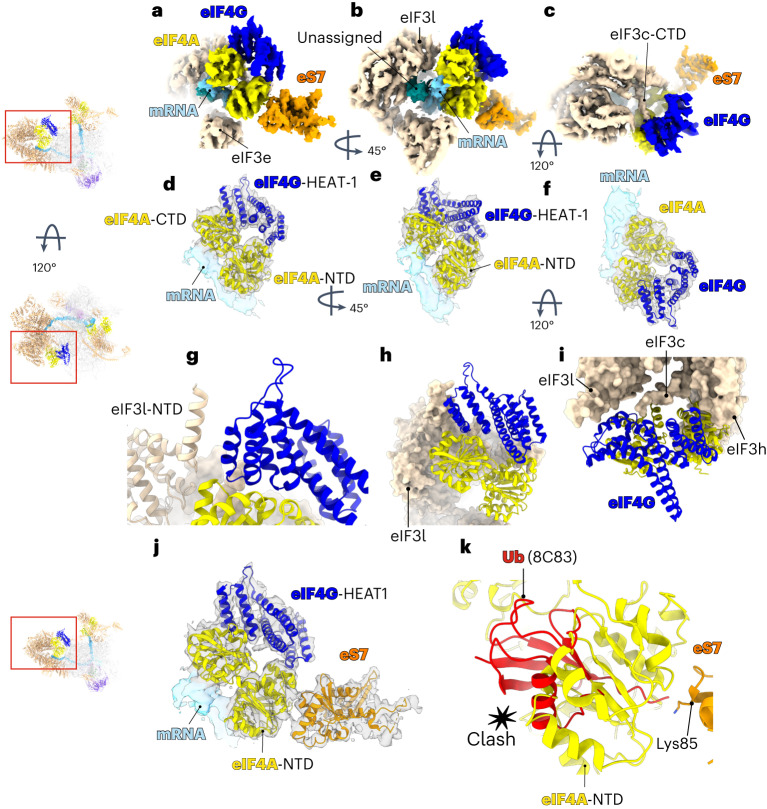
Structure of eIF4F and its interactions with eIF3 and ribosomal protein eS7. **a**–**c**, Cryo-EM map to highlight eIF4F and its interaction network shown in in different orientations. **d**–**f**, Atomic model of human eIF4A and eIF4G fitted into the cryo-EM map shown in in different orientations. **g**–**i**, The insets highlight the interaction network of eIF4F with eIF3. **j**, Close-up to highlight the interaction between eIF4F and ribosomal protein eS7. **k**, Superposition of ubiquitin (PDB:8C83)^53^ with the structure of human 48S to highlight the possible clash between eIF4A and ubiquitin at its predicted binding site (Lys85).

**Extended Data Fig. 7:**
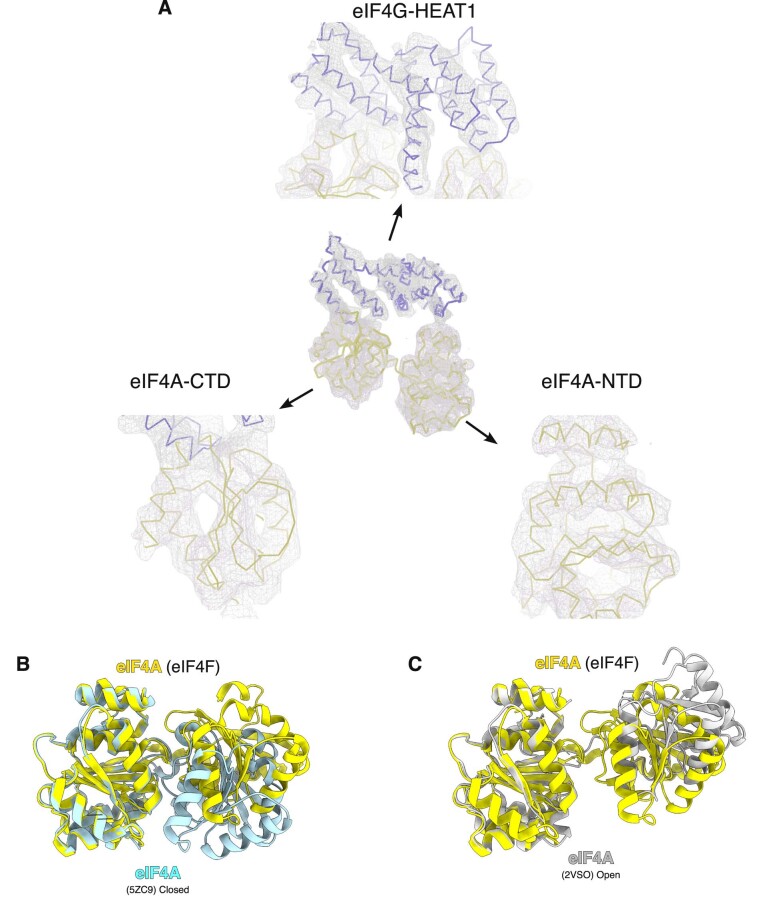
Human eIF4F. (**a**) Atomic model of human eIF4F (eIF4G and eIF4A) fitted into the cryo-EM map after focus refinement on eIF4F. (**b**) Superposition of eIF4A that is part of the cap-binding complex in the 48S with the structure of human free eIF4A in complex with mRNA and RocA (PDB: 5ZC9)16. (**c**) Superposition of this eIF4A with the structure of a yeast eIF4A in complex with eIF4G (PDB: 2VSO)23.

The density shows almost the entire secondary structure of the middle domain of eIF4G (HEAT-1) and eIF4A (Fig. 4a–c and Extended Data Fig. 7a). Rigid-body fitting of a crystal structure of a human eIF4A (Protein Data Bank (PDB): 5ZC9)^16^ allowed us to assign its corresponding density (Fig. 4d–f). A model of the middle domain of eIF4G based on an AlphaFold prediction^37^ was used for rigid-body fitting into the density (Fig. 4d–f and Extended Data Fig. 7a). The predicted model agreed well with the density, allowing us to locate the domain of eIF4G in the structure.

eIF4F is positioned on the 5′ side of mRNA relative to the 40S subunit, near the mRNA channel exit site. For the first time, mRNA interacting with eIF4A is clearly visible (Fig. 4a–c), and eIF4A adopts a structure intermediate between the extended conformation, found in a crystal structure of a yeast eIF4A–eIF4G complex without mRNA in which the two RecA domains are rotated away from each other^23^, and the closed conformation of an eIF4A–mRNA dimeric complex^16^. This conformation allows the two RecA domains to interact with the mRNA while maintaining their interactions with eIF4G (Fig. 4a–f and Extended Data Fig. 7b,c).

In metazoans, eIF3 plays a critical role during the recruitment of 43S to the eIF4F–mRNA^3–5,8,10^. The structure reveals an unexpectedly large interaction network between eIF3 and eIF4F (Fig. 4g–i). Previously, because of high flexibility, the structure of eIF3l-NTD remained elusive. The improved local resolution reveals a near-complete structure of eIF3l-NTD and its interaction with eIF4G. Interestingly, the interaction between eIF3l-NTD and eIF4G occurs through a domain of eIF4G located between residues Met775 and Ala787, which was unexpected (Fig. 4g). Additionally, the cryo-EM map reveals an additional density connecting eIF4G with eIF3c and eIF3h (Fig. 4). Although the local resolution does not allow us to assign all the side chains in the density, it probably belongs to the C-terminal tail of eIF3c (Fig. 4c). The interaction between eIF3c and eIF3-binding domain of eIF4G was previously predicted by site-specific cross-linking^3^. The structure shows that this interaction occurs through the same eIF3-binding domain of eIF4G that is involved in the interaction with eIF3l (Fig. 4c,g).

**Extended Data Fig. 8:**
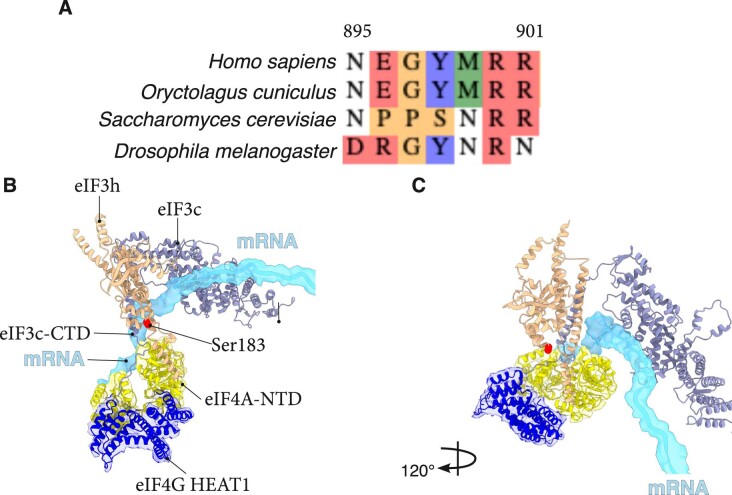
Multiple sequence alignment and Interaction of eIF3 with eIF4F. (**a**) Multiple sequence alignment of the eIF3C C-terminal tail. The site of interaction with eIF4G is conserved among eukaryotes. (**b**, **c**) Interaction of eIF3h and eIF3c with eIF4F. eIF3h interacts directly with eIF4A-NTD. eIF3h and eIF3c provide clamping and guide the mRNA (cryo-EM map) from eIF4F towards the mRNA channel in the 40S.

Previously, eIF4F was observed to interact with the 43S complex through the eIF3 subunits -e, -k and -l^4^. However, these subunits are not present in *S. cerevisiae* eIF3, and eIF3k and eIF3l are dispensable in *Neurospora crassa* and *Caenorhabditis elegans*^38,39^. Thus, these previously identified interactions could not occur in all eukaryotes, raising the question of how eIF4F makes an interaction with the 43S in those species. In this higher-resolution structure, we see several additional interactions between eIF4F and the 43S complex that are likely to be universally conserved. One of these could be the eIF4G–eIF3c interaction, given that the eIF3c C-terminal tail includes residues highly conserved among eukaryotes, including in *S. cerevisiae* (Extended Data Fig. 8a), which also explains why a knockdown of eIF3c reduces the recruitment of the 43S^40^.

The eIF4A component of eIF4F binds to a pocket formed by eIF3c, eIF3e, eIF3h and eIF3l (Fig. 4). We previously described interactions between eIF4A and eIF3e, and eIF3k/eIF3l^4^. The improved local resolution of this post-scanning complex allowed us to further characterize the interaction between eIF4A and the octameric structural core of eIF3. Of the two RecA domains of eIF4A, the C-terminal domain (CTD) is in close proximity to eIF3l, while the NTD makes contact with eIF3c, eIF3e and eIF3h.

The interactions between eIF4A and eIF3h raise many questions. eIF3h is known to interact with METTL3 and promote mRNA circularization during m^6^A-dependent translation^41^. The two helices from eIF3h extend toward the solvent site of the 43S, but the role of eIF3h in initiation was not clear. The core of the mammalian eIF3 octameric structural core is formed by a seven-helix bundle consisting of two helices from eIF3h and one helix from eIF3c, eIF3e, eIF3f, eIF3l and eIF3k^4,42–45^. Here we see a direct interaction between eIF3h and eIF4A-NTD (Fig. 4i and Extended Data Fig. 8b,c) through which eIF3h may play a regulatory role in initiation (Fig. 4). Consistent with this idea, phosphorylation at residue Ser183 of eIF3h, located just above the domain that interacts with eIF4A (Extended Data Fig. 8), has been implicated in the process of reinitiation in plants^46^. In humans, the same phosphorylation has been associated with a possible oncogenic role of eIF3h^47^. It is possible that this post-translational modification of eIF3h affects its interaction with eIF4F. Furthermore, this domain of eIF3h also interacts with mRNA (Extended Data Fig. 8).

Unexpectedly, the structure reveals a direct interaction between eIF4A-NTD and ribosomal protein eS7 (Fig. 4j). The structure rationalizes previous studies on translational control whereby eS7 is monoubiquitinated by the human E3 ubiquitin (Ub) ligase CNOT4 or its yeast ortholog Not4 (refs. ^48–51^). Deubiquitination is required to allow the cap-binding complex eIF4F to bind to the 43S^52^. Here we see a direct interaction between eIF4A-NTD and eS7 (Fig. 4j,k). Superimposing our structure with a structure of Ub^53^ at the predicted binding site in eS7 (refs. ^52,53^) places the Ub in a position where it could clash with eIF4A (Fig. 4k), thereby preventing the binding of the cap-binding complex eIF4F, which suggests why deubiquitination of eS7 may be required for the binding of eIF4F^52^. However, translation can still initiate through eIF4F-independent mechanisms^53^.

The cryo-EM map also reveals some additional density between the mRNA and eIF3l (Fig. 4a,b). A portion of it resembles the shape of an A-form double helix, suggesting that a portion of the 5′ UTR forms a stem loop structure, but other parts resemble a protein tail and at this resolution, it was not possible to interpret it unambiguously. It is worth noting that eIF4F was present in the same location in a 48S complex assembled on an mRNA with β-globin 5′ UTR, (Extended Data Fig. 4), and also in our previous structure of a 48S complex using an mRNA with an unstructured 5′ UTR^4^. Thus, the location of eIF4F is not dependent on the unassigned density present here. It is possible that this yet uncharacterized structure may confer additional stability to eIF4F beyond that provided by its interactions with eIF3 and eS7 as well as affect its precise orientation.

## Upon start-codon recognition, human eIF5-NTD replaces eIF1

**Extended Data Fig. 9:**
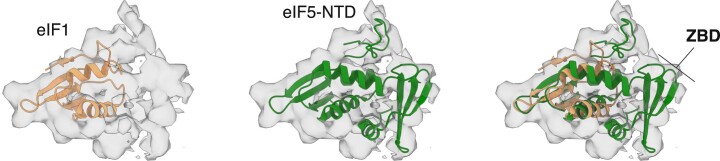
Map-to-model fits of human eIF1 and eIF5. Rigid-body fitting of human eIF1 (PD- B:6ZMW)^4^ and eIF5-NTD (PDB: 2E9H) into the cryo-EM map. Zinc binding domain (ZBD).

Around the site normally occupied by eIF1, there is additional density that cannot be accounted for by the factor (Fig. 1, Extended Data Fig. 9). Both previous biochemical data and a structure of a yeast initiation complex showed that the yeast NTD of eIF5 (eIF5-NTD) replaces eIF1 upon start codon recognition^54,55^. Rigid-body fitting of the known structure of human eIF5-NTD (PDB: 2E9H) accounted for the entire density present at the platform of the 40S (Extended Data Fig. 9). Moreover, we could fit the zinc-binding domain of eIF5-NTD into the density (Extended Data Fig. 9). This domain is not present in eIF1, confirming that this density arises from bound eIF5-NTD that has displaced eIF1 upon start-codon recognition just as was previously proposed for the yeast system^54,55^.

**Fig. 5:**
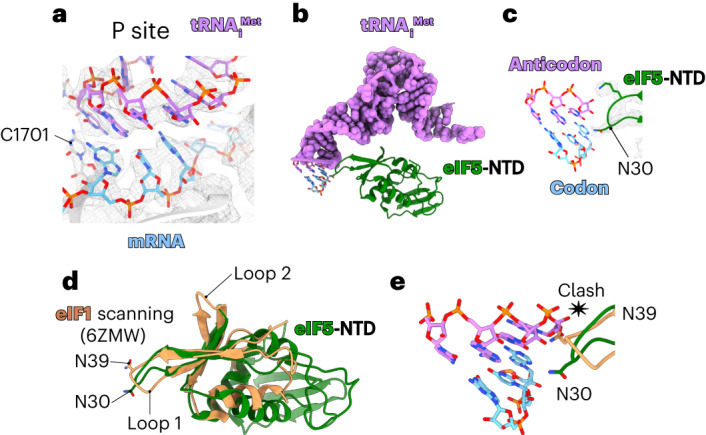
Codon–anticodon base pairing in the P site. **a**, Close-up of the P site to highlight the codon–anticodon base pairing. **b**, eIF5-NTD replaces eIF1 after AUG start codon recognition. **c**, Close-up view illustrating the interaction between eIF5-NTD, tRNA, and mRNA in the P site. **d**, Superposition of eIF5-NTD with the structure of human eIF1 during scanning (PDB: 6ZMW)^4^. **e**, The conformation of eIF1 during scanning would clash with the anticodon stem loop of the tRNA_i_^Met^ after start codon recognition.

The structure therefore reveals an evolutionarily conserved set of events that occur on start-codon recognition, whose fidelity is enhanced by the factors eIF1, eIF1A and eIF5 (ref. ^56^). First, eIF1A and eIF1 bind to the 40S platform near the A and P sites, respectively, and promote the open conformation of the 40S that facilitates the binding to the tRNA_i_^Met^. During scanning, the tRNA_i_^Met^ is not fully inserted into the P site of the ribosome^4^, which allows codon–anticodon sampling until recognition of the AUG start codon^4,57^. After start-codon recognition (Fig. 5a), the tRNA_i_^Met^ is inserted fully into the P site of the ribosome^42,43,57–61^, resulting in a clash with eIF1. Thus, upon start-codon recognition, eIF1 dissociates from the complex and is replaced by eIF5-NTD (Fig. 5). Like eIF1, eIF5-NTD monitors the codon–anticodon interaction (Fig. 5b,c). However, while eIF1 residue Asn39 clashes with the anticodon stem-loop, the eIF5-NTD residue Asn30 adopts a different conformation (Fig. 5d,e), which allows it to monitor codon–anticodon interaction without clashing with the tRNA_i_^Met^ (Fig. 5c).

During scanning, eIF1 Loop 2 interacts with the tRNA_i_^Met^ D loop, which prevents the accommodation of the tRNAi^Met^ into the P_IN_ state. Upon start codon selection, Loop 2 undergoes conformational changes to allow the accommodation of the tRNA_i_^Met^ in the P_IN_ state^57,62^. The destabilization of eIF1 and its replacement with eIF5-NTD avoids a clash with the tRNA_i_^Met^ and stabilizes it in the P_IN_ state because eIF5-NTD Loop 2 is both shorter and adopts a different conformation than that of eIF1 (Fig. 5d).

## Discussion

**Fig. 6:**
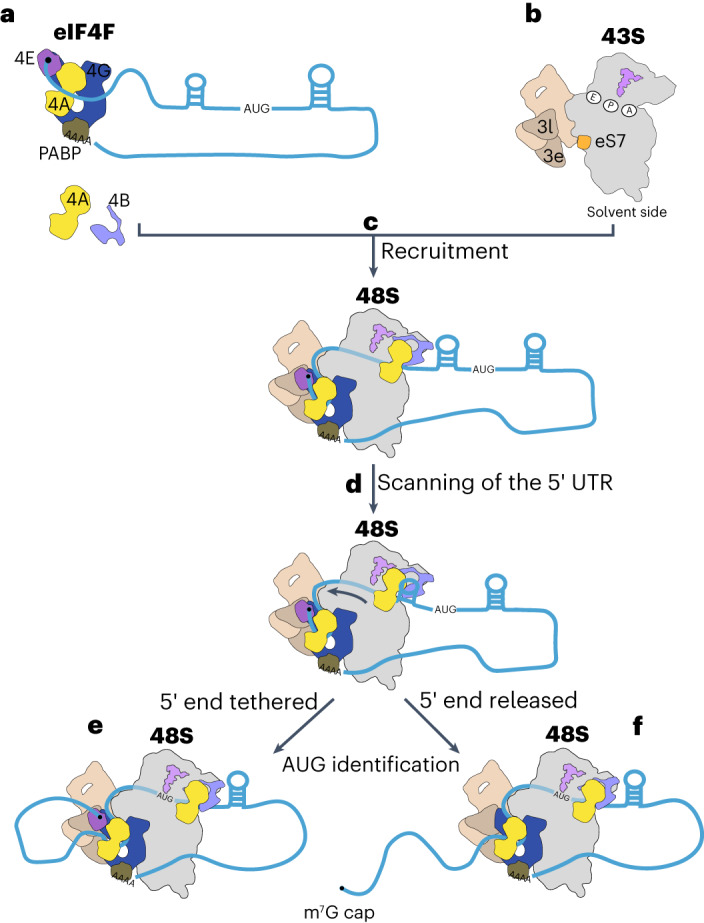
Model for translational regulation by eIF4F and eIF4A. **a**–**c**, The cap-binding complex eIF4F (**a**) binds at the 5′ end of mRNA and recruits the 43S (**b**) to form the 48S complex (**c**). The fuzzy line represents the mRNA into the channel in the 40S. eIF4F binds to eIF3 and ribosomal protein eS7 located upstream of the 43S. The position of eIF4F is compatible with a model whereby the mRNA is slotted into the mRNA channel in the 40S small ribosomal subunit. In addition to eIF4F, a second molecule of eIF4A at the mRNA entry site is likely to play a role in this process by facilitating the accommodation of the mRNA into the channel. The ATPase activity of eIF4A is stimulated by eIF4B and eIF3bgi module 15 located at the entry site. **d**, Thus, eIF4A at the entry site is likely to use the energy from ATP hydrolysis to unwind the RNA secondary structure downstream of the 43S. **e**,**f**, It remains unclear whether eIF4E remains attached to the rest of eIF4F (**e**) or is released (**f**) during the scanning process, but the structure suggests the latter.

In our structure, the cap-binding complex eIF4F binds upstream of the 43S complex, while a second molecule of eIF4A binds downstream, at the entry site of the mRNA binding channel. These locations suggest a model where eIF4A plays eIF4F-dependent and eIF4F-independent roles during mRNA recruitment^15^ and scanning (Fig. 6a–c).

The cap-binding complex eIF4F at the 5′ end of mRNA plays a critical role in recruiting the 43S downstream of it, in a process that involves the mRNA being slotted into its channel in the 40S subunit (Fig. 6c). The higher resolution of the current 48S complex reveals how eIF4F makes universally conserved interactions with the 48S complex, including eIF3c and ribosomal protein eS7.

Canonical eIF4F-dependent initiation is enhanced by a topologically closed loop of the mRNA, often mediated by an interaction between eIF4G at the 5′ end and PABP at the 3′ end^63,64^. Such a topology would require a slotting mechanism for recruitment. Similarly, during m^6^A-dependent translation, it has been shown that the mRNA is circularized through an interaction between METTL3 located at the 3′ of mRNA and eIF3h^41^. Other modes of initiation also require the slotting mechanism, including that via internal ribosome entry sites, eIF3d-dependent or circular mRNA. Some mRNAs with unusually short 5′ UTR (less than 40 nucleotides in mammals) may use recruitment pathways other than slotting. A recent study proposed that these mRNAs may use the slotting mechanism as well^65^, which would require a backward movement of the 43S (3′ to 5′) rather than the small oscillations that are known to exist^22^. Thus, it is more likely that those mRNAs use an alternative recruitment pathway.

**Extended Data Fig. 10:**
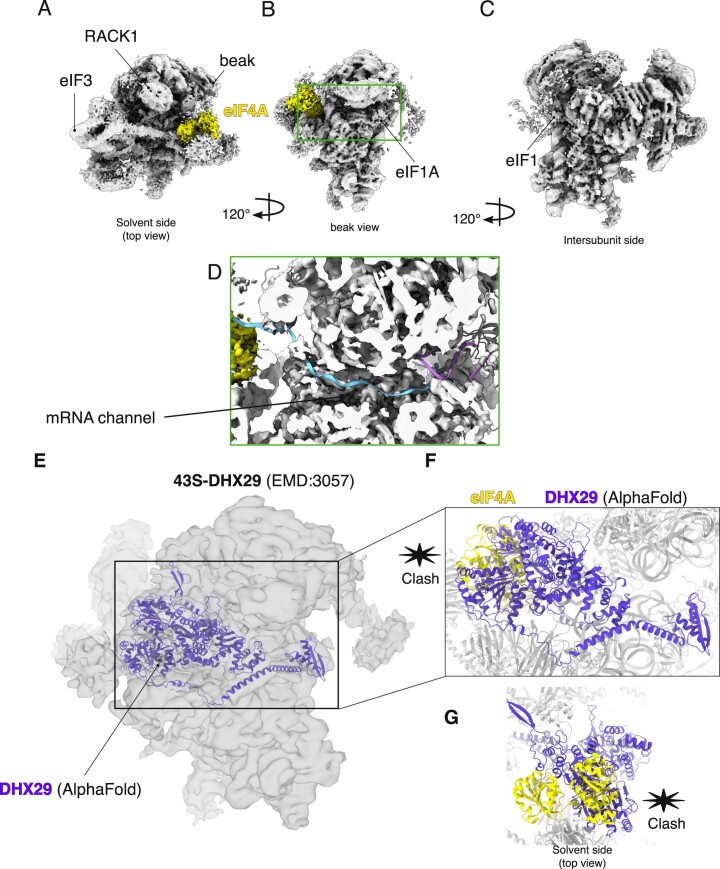
Cryo-EM 3D reconstruction of a class of particles without mRNA into the channel and the position of eIF4A. (**a** to **c**) Overview of the cryo-EM density map of a class of particles without mRNA into the channel. (**d**) The inset shows a lack of density for mRNA using mRNA and tRNA superimposed from the 48S structure. (**e**) Rigid-body fitting of human DHX29 (AlphaFold prediction) into a prior cryo-EM map of a rabbit 43S (EMD:3057)^44^. (**f** and **g**) The inset shows that DHX29 and the entry-site eIF4A would overlap and could not coexist.

In addition to eIF4F, a second eIF4A helicase is coordinated by the 40S subunit and eIF3 so that it binds to mRNA before it enters the mRNA binding channel (Fig. 6c). The function of this entry-site-bound eIF4A may promote the accommodation of the mRNA into the entry channel, especially when secondary structure is present in the 5′ UTR^15,66^. Consistent with this idea, the cryo-EM 3D classification reveals a class of particles with eIF4A bound to the 43S but without the mRNA accommodated into the channel at the entry site (Extended Data Fig. 10a–d), suggesting that the binding of the second eIF4A to the 43S precedes the binding of the mRNA. Given the possible location of the eIF4B-RRM in our structure, it is possible that eIF4B may also play a role in the mRNA accommodation process^67^.

The position of eIF4A at the entry site of the mRNA binding channel provides strong evidence that this conserved RNA helicase probably functions to unwind the mRNA secondary structure as it enters the mRNA binding channel during scanning. Thus, we propose a model whereby after recruitment, both eIF4A and eIF4F work in concert to ensure the directionality of scanning (Fig. 6d). In this model, eIF4F would prevent the reverse movement of the 43S complex^4^ while the second molecule of eIF4A at the leading edge of the ribosome would use the energy from ATP hydrolysis to unwind downstream RNA secondary structure and translocate along the 5′ UTR of mRNA during scanning (Fig. 6d). It is possible that ATP hydrolysis by eIF4F might further help the movement of the 43S and help overcome energy barriers encountered by the entry channel eIF4A.

Compared to many other helicases, eIF4A has low helicase activity. Even though its ATPase activity is stimulated by the 43S and its cofactor eIF4B, scanning of 5′ UTR of mRNA with highly stable secondary structure may require additional helicases, such as mammalian DHX29, which has been seen to bind at the mRNA channel entry site^44,68^. In our structure, eIF4A–eIF4B interacts with the 43S at the exact location that DHX29 does^44,68^ (Extended Data Fig. 10e–g). Thus, it is possible that, for initiation on specific mRNAs with high secondary structure, other helicases such as DHX29 are recruited to the 43S complex instead of a second eIF4A molecule. Such a method would allow additional control of translation initiation of such mRNAs.

eIF4E stabilizes the binding of mRNA to eIF4G–eIF4A^36^ and stimulates the helicase activity of the eIF4F bound eIF4A^69^. Selective 40S ribosome profiling has indicated that scanning is cap-tethered in most human cells^70^ (Fig. 6e). In the prior structure of 48S there is a low resolution density that we tentatively assigned to eIF4E^4^. That structure used a very short mRNA, so no meaningful scanning had occurred. In the current structure, scanning has spanned 105 nucleotides before the start codon was reached, thus representing a later stage of initiation. Although local resolution was improved, we did not see such additional density that we could assign to eIF4E, suggesting that it, along with the 5′ cap, is released from eIF4F and the rest of the 48S during scanning, a possibility that has been previously suggested^71,72^ (Fig. 6f).

In conclusion, our structural data reveal how two eIF4A helicase proteins coordinate the binding of mRNA to the entry and exit sites of 40S subunit mRNA binding channel. Importantly, the entry-channel-bound eIF4A helicase is precisely positioned to enable its ATPase activity to directly unwind secondary structure located downstream of the scanning 48S complex. Our suggested location of eIF4B adjacent to this second eIF4A molecule would explain its role in the helicase activity of eIF4A. The eIF4A helicase has become an important therapeutic target, with eIF4A binding natural products showing promising anti-tumor activity in preclinical studies. The discovery that two copies of eIF4A are bound to the 48S complex may therefore help guide strategies for cancer therapy.

## Methods

### Purification of human eIFs

Ribosome, tRNA_i_^Met^ and human eIFs, including eIF4G_557–1,105_, were purified as described previously^61,66,69,73^. Briefly, native eIF2, eIF3, and 40S small ribosomal subunits were purified from HeLa cell extracts. Recombinant eIF1, eIF1A, eIF5, eIF4E and eIF4E were expressed in BL21 (DE3) cells and purified using a combination of affinity and size exclusion liquid chromatography. Recombinant eIF4G was expressed using the baculovirus-insect cell (sf9) expression system. Finally, PABP was purified as described^69^. The protein was expressed in BL21 bacterial cells using maltose-binding protein (MBP) fusion constructs. This construct was designed to include a tobacco etch virus protease cleavage site, enabling the removal of the tag. Subsequently, the recombinant PABP–MBP fusion protein was subjected to incubation with tobacco etch virus protease, facilitating the cleavage of the tag, and underwent additional purification steps to isolate the untagged PABP. Additionally, we used a hydroxyapatite column to remove any remaining nucleic acids bound to it, and the protein was purified further by gel filtration chromatography on a Superdex 200 Increase column in a buffer containing 20 mM HEPES, pH 7.5, 200 mM KCl, 10% glycerol and 1 mM tris(2-carboxyethyl)phosphine.

### In vitro transcription and purification of mRNA

We designed a synthetic DNA containing a T7 promoter followed by a 5′ UTR containing three polypurine motifs (GGACAAGAGAGAGAGAGACUCCAACUCCAAGAGAGAGAGAGA

CAACUCCAAGAGAGAGAGAGACAAACCCUCGCUGAGCCGCAGUCAGAUCCUAGCGUCGAGUUG**AUG**CUGUCCGAU). The construct was purchased as a gene block from IDT. After linearization, the DNA plasmid was used as a template for in vitro transcription. The mRNA was purified using a denaturant acrylamide gel (8 M urea) followed by electroelution. The purified mRNA was capped using Vaccinia Capping System (New England Biolabs) and then polyadenylated (~90 nucleotides) (Extended Data Fig. 1) using *Escherichia coli* Poly(A) polymerase (New England Biolabs). After free nucleotide, enzyme and buffer removal, the pure mRNA was stored in water.

We used the same protocol of transcription and purification of DLP mRNA containing β-globin 5′ UTR used for cryo-EM

(GACACUUGCUUUUGACACAACUGUGUUUACUUGCAAUCCCCCAAAACAGACAGA**AUG**AACAACGAGCCACCGCAAACCAGUGACGGCCGCCGGAGGCGCCCGCGCCCGGCGGCCGAGAGAGCAGAACGAGACCACACGGAUCCGAGAAGAUUCAUCCUCCUUCAAUGCCUGGAGGAUA).

### In vitro reconstitution of human 48S complexes for cryo-EM

We reconstituted the 48S complex by mixing the 43S with eIF4F, eIF4A, eIF4B, PABP and mRNA in a 25 μl reaction. To reconstitute the 43S, we mixed 0.5 μM 40S with 0.9 μM eIFs and 1.8 μM TC to a final volume of 18 μl in a buffer (20 mM HEPES–KOH pH 7.5, 97 mM KAc, 2.5 mM MgAc, 3% glycerol, 0.1 mM spermidine, 1 mM dithiothreitol and 0.5 mM GMP-PNP). We incubated the 43S reaction mix at 30 °C for 10 min. In parallel, we assembled the cap-binding complex eIF4F by mixing a co-purified eIF4G (residues 165–1,599)–eIF4A complex with eIF4E, mRNA and PABP, to a final concentration of 3.3 μM in 12 μl reaction in a buffer (20 mM HEPES–KOH pH 7.5, 97 mM KAc, 2.5 mM MgAc, 3% glycerol, 0.1 mM spermidine, 1 mM dithiothreitol and 0.5 mM ATP-g-S). Finally, we mixed 1 μM eIF4F, 1 μM eIF4B, 1 μM eIF4A, 43S (0.3 μM 40S and 0.5 μM eIFs) and 0.5 mM RocA in a 25 μl reaction. The reaction mix was incubated at 30 °C for 10 min.

The same protocol was used to assemble the 48S complexes with eIF4G_557–1,105_, without eIF4B and with DLP mRNA. The DLP 48S was assembled without RocA.

### Cryo-EM grid preparation

The assembled 48S complexes were cross-linked using 1.5 mM BS3 (final concentration) on ice for 45 min to prevent dissociation of eIFs during the grid preparation. As described before^4^, the cross-link reaction in the presence of spermidine (a polyamine with quenching properties) and on ice has a mild effect—comparable with the complex without BS3. Three microliters of 140 nM 48S complex (based on the concentration of the 40S) was applied onto UltrAuFoil R1.2/1.3 300 mesh gold grids precovered with graphene oxide (Sigma) suspension made in-house. Briefly, the UltrAuFoil gold grids were washed using deionized water and then dried overnight at room temperature. The grids were then glow-discharged for 5 min at 30 mA, followed by incubation with 3 μl of graphene oxide (0.2 mg ml^−1^) for 1 min. After the incubation and blotting, the grids were washed three times using 20 μl deionized water. The grids were dried for at least 1 h at room temperature and then used.

We prepared the grids using an FEI Vitrobot Mark IV at 4 °C and 100% humidity. The grids were blotted for 7 or 8 s at blotting force −15 and then plunged into liquid ethane at 93 K in a precision cryostat system produced at the MRC Laboratory of Molecular Biology^74^.

### Cryo-EM data collection

Data were collected on Titan Krios microscopes (ThermoFisher) equipped with a K3 direct electron detector camera (Gatan) at a magnification of 105,000× and at pixel sizes of 0.826 Å per pixel or 0.829 Å per pixel (48S dataset from eBIC/Diamond). The data were collected using EPU software, super-resolution counting mode using a Bio-quantum energy filter (Gatan) (binning 2), faster acquisition mode, and with defocus ranging from −1.2 μm to −3.0 μm.

The following doses/frames were used: 1.197 e^−^ Å^−^^2^ (48S dataset from eBIC/Diamond); 1.2391 e^−^ Å^−^^2^ (48S dataset from MRC LMB); 0.6889 e^−^ Å^−^^2^ (48S without eIF4B and 48S complexes with eIF4G_682–1,105_). DLP 48S was collected using Falcon 4 electron detector, counting mode and 1 e^−^ Å^−^^2^ per frame.

### Image processing

Motion correction was performed using the implementation in RELION 4 (ref. ^75^). Movies were aligned using 5 × 5 patches with dose weighting. CTF (contrast transfer function) was estimated using CTFFIND4.1 (ref. ^76^). After 2D classification, we used the cryo-EM map of a human 48S^4^ after low-pass filtering to 60 Å as a reference for 3D classification. After 3D classification and refinement, we performed mask classification to select only particles containing eIF3 octameric structural core. We used Bayesian polishing in RELION to correct beam-induced motion^75^. After polishing, we performed mask classification at the entry site, followed by the eIF4F binding region and TC. Finally, we used multi-body refinement and flexibility analysis in RELION^77^ to account for the predominant molecular motions.

### Model building, fitting and refinement

We used our previous structure of human 48S^4^, previous cryo-EM and crystal structures of eIFs^16,23,25,26,42,44,54,60,78–80^, as well as AlphaFold^37^, to build and refine the atomic model. Fitting and model building and local refinement were performed in Coot^81^. We used Phenix for real space refinement^82^.

### Figures

All figures were made using ChimeraX^83^.

### Reporting summary

Further information on research design is available in the Nature Portfolio Reporting Summary linked to this article.

## Online content

Any methods, additional references, Nature Portfolio reporting summaries, source data, extended data, supplementary information, acknowledgements, peer review information; details of author contributions and competing interests; and statements of data and code availability are available at 10.1038/s41594-023-01196-0.

### Supplementary information

Reporting Summary

## Data Availability

The atomic model and cryo-EM maps have been uploaded to the Protein Data Bank (PDB: 8OZ0) and to the Electron Microscopy Data Bank (EMD: 17297). Maps of 3D focus refinement and 3D multi-body have also been uploaded to EMDB under the same accession number (EMD: 17297).
